# Feeding Deterrence to *Reticulitermes speratus* (Kolbe) by *Fibroporia radiculosa* (Peck) Parmasto 1968

**DOI:** 10.3390/insects8010029

**Published:** 2017-03-06

**Authors:** Nadia Nuraniya Kamaluddin, Shigeru Matsuyama, Akiko Nakagawa-Izumi

**Affiliations:** Graduate School of Life and Environmental Science, University of Tsukuba, Tsukuba, Ibaraki Prefecture 305-8571, Japan; nadia.kamaluddin@gmail.com (N.N.K.); matsuyama.shigeru.fu@u.tsukuba.ac.jp (S.M.)

**Keywords:** *Fibroporia radiculosa*, feeding behavior, *Reticulitermes speratus*

## Abstract

Brown rot fungus *Fibroporia radiculosa* (Peck) Parmasto grown in decayed wood and non-wood material, potato dextrose agar (PDA) media, deterred *Reticulitermes speratus* (Kolbe) feeding. Decayed wood and PDA media were extracted and tests were performed to assess termite feeding behavior towards the extracts. We found that the extract from PDA media also suppressed termite feeding, although it did not induce mortality. Using gas chromatography and mass spectrometry analysis, two bioactive compounds were detected from the decayed wood extract, and one was detected from the PDA extract. Based on National Institute of Science and Technology (USA) Mass Spectral library match and compound fragmentation, both of the compounds belong to the sesquiterpenes family.

## 1. Introduction

Both higher and lower termites interact with fungi [1]. In particular, termites and wood-decay fungi both decompose woody substances; therefore, several forms of interactions likely formed between these two organisms. For example, non-entomopathogenic fungi can influence the palatability of wood [2,3], acting as feeding attractants, stimuli or preference inducers [4,5], or as deterrents [6] towards termites. Thus, fungi-termite interactions are ubiquitous, although they are sometimes provisional in character and cannot be categorized as symbiosis [7].

Factors such as fungal strain, degree of decay, as well as tree and termite species, should be considered in fungi-termite interactions, especially with respect to termite feeding preferences for fungus-decayed wood [1,2]. Wood-decay fungi facilitate termite consumption of lignocellulose [8] through the degradation or modification of wood components. Wood-decay fungi trigger various responses in termite feeding behavior under field and laboratory conditions [1,4,8]. For instance, fungal chelate accumulation during wood decay initiates lignin oxidation, cellulose depolymerization, and lignin degradation reactions, which may act as chemosensory signals to termites that the wood is heavily degraded and therefore nutritionally inadequate [6]. However, wood-decay fungi also appear to detoxify wood extractives, rendering soft wood more suitable for termite consumption [5].

Although interactions between termites and decay fungi are frequently observed on wood as a fungal substrate, it remains unclear whether these interactions are actually mediated by the presence of wood components or solely between the termite and fungus themselves. Therefore, it is important to clarify whether termite feeding is related to fungal secretion or products released during wood degradation. In our previous study, we reported a feeding deterrent effect from stakes decayed by brown rot basidiomycete *Fibroporia radiculosa* [9]. We determined that the compounds linked to this deterrent effect are also present in *n-*hexane wood extracts; both field-decayed wood stakes and laboratory-decayed wood extract yielded the same outcome. This study aimed to further verify the identity and origin of the compound from *Fibroporia radiculosa* (Peck) Parmasto that is responsible for the feeding deterrent effect on *Reticulitermes speratus* (Kolbe).

## 2. Materials and Methods

### 2.1. Termites

Five *R. speratus* colonies were used in this study: three colonies (Oarai 1, 2, and 3) were obtained from Oarai, Ibaraki Prefecture, Japan, during October 2016. Two colonies (Kagoshima 1 and 2) were obtained from a field maintained by the Research Institute for Sustainable Humanosphere (RISH) in Kagoshima Prefecture, Japan, during December 2014. All termite colonies were housed in plastic containers in a temperature- and moisture-controlled laboratory until use. Different colonies were used for each test; no colony was used more than once.

### 2.2. Pure Cultures of Fibroporia radiculosa

*Fibroporia radiculosa* cultures were previously isolated, identified, and purified from decayed stakes obtained from a RISH field in 2009 [9]. The fungus was maintained in a potato dextrose agar (PDA) slant containing 100 ppm benlate and 50 ppm tetracycline to prevent mold growth, and kept under refrigerated conditions (2 to 3 °C) until use.

### 2.3. Preparation of Decayed Wood and PDA Media

Laboratory decayed woods were prepared by drying block samples from sapwood of *Pinus densiflora* (20 mm × 20 mm × 5 mm) at 60 °C for 48 h, followed by sterilization by ethylene oxide gas. Three sterilized woods were aseptically placed on a *F. radiculosa* mycelium grown in a diluted potato dextrose agar medium and kept inside an incubator at 27 °C for eight weeks. To prepare diluted Potato Dextrose Agar (PDA) medium for laboratory decayed wood, 1.3 g of PDA powder (Nissui Pharmaceuticals, Tokyo, Japan) and 2.6 g of agar (Nacalai Tesque, Kyoto, Japan) were diluted in 100 mL tap water. The medium was poured into a 500 mL screw-capped bottle and sterilized by autoclaving at 121 °C for 15 min. Sterilized medium was inoculated with *F. radiculosa* pure cultures and incubated at 27 °C for seven days or until the mycelium has covered the diluted PDA surface.

Meanwhile, decayed PDA medium were prepared via mixing 3.9 g of PDA powder (Nissui Pharmaceuticals, Tokyo, Japan) with 100 mL of tap water, then poured into a 500-mL screw-capped bottle for autoclaving at 121 °C and 15 min. Pure culture of *F. radiculosa* were transferred aseptically to the media surface. Cultured medium were cultivated in a 27 °C incubator for eight weeks.

### 2.4. Extract Preparation of Extract and PDA Media

Decayed *P. densiflora* blocks were removed from the incubation bottle. Blocks surface were carefully cleaned from fungal mycelium. The woods were freeze-dried overnight, cut into smaller pieces, and ground using a mortar and pestle to obtain decayed wood powder. Five grams of the decayed wood powder was inserted into a 200 mL conical flask and subjected to extraction with 100 mL of *n-*hexane at 18–25 °C overnight (10–12 h). The mixture was filtered using filter paper (No. 1, Advantec Toyo Roshi, Tokyo, Japan) and concentrated using a rotary evaporator into a 10 mL solution.

For extract preparation from fungal cultures cultivated on growth media, the mycelium-covered medium surface was carefully scraped using a spatula; the collected material was ground with a glass stirrer, and then added to a 300 mL conical flask. The ground medium (100 g) was extracted with 100 mL of *n-*hexane overnight or up to 24 h. The mixture was filtered using No. 1 filter paper (Advantec Toyo Roshi) and concentrated to 5 mL in a rotary evaporator at 10 °C, then refrigerated (2–3 °C) for a maximum of 14 days after extraction.

### 2.5. Purification of Extract

The decayed wood and PDA extracts were purified using small-scale column chromatography. Into a cotton wool plugged Pasteur pipette (146 mm, Corning, New York, NY, USA) silica gel (C200, Wako Pure Chemicals Industry, Osaka, Japan) was dry-packed (4 cm long). Then an aliquot of 4 mL of extract was added; *n-*hexane, diethyl ether, and ethyl acetate were used. Each portion was divided into two equal parts for bioassay and chemical analyses using gas chromatography/mass spectrometry (GC/MS).

### 2.6. Feeding Deterrent Bioassays (No-Choice and Two-Choice Feeding Tests)

#### 2.6.1. No-Choice Feeding Test of Crude and Purified Extract from Wood and PDA Media Decayed by *F. radiculosa*

No-choice feeding tests were conducted as follows: paper disks (Ø 8 mm, thick type; Advantec Toyo Roshi) were soaked with 100 μL of decayed wood extract (containing 50 mgeq (milligrams equivalent) of decayed wood), 100 μL of decayed PDA extract (containing 500 mgeq of decayed PDA media), or 100 μL of sound wood extract (containing 5 mgeq of sound wood) used as control. The disks were then treated with 100 μL of *n*-hexane were used as a solvent treatment. Treated paper disks were placed inside a fume hood for 15 min to remove the solvent before being transferred into individual plastic saucer (Ø 1 cm) on top of 15-mm-thick hard plaster (New Plastone Dental Stone, GC Corp., Tokyo, Japan). The top of hard plaster was covered with a thin layer of wet sand on which a sample disk on the saucer was placed. The bottom of each cup was perforated (perforations 10 mm in diameter) to supply moisture through the plaster layer, which was in contact with a damp paper pad at the bottom of the test chamber. Fifty *R. speratus* workers of Kagoshima 1 colony were introduced to each cup. Test chambers were kept inside an incubator for five days at 25 °C and 70%–80% relative humidity (RH). Seven biological replicates were performed (seven cups each with 50 *R. speratus*) for each treatment. At the end of the test period, paper disks were weighed to assess mass loss from termite feeding and the remaining termites were counted to determine the mortality percentage.

Purified extracts bioassay was conducted in a similar manner to crude extracts, but with 300 μL (150 mgeq decayed and sound wood, 1500 mgeq of decayed PDA) of purified extracts. The tests were conducted for four days at 25 °C and 70%–80% RH, and replicated five times (five cups of 50 *R. speratus* for each treatment) using Oarai 3 colony. In no-choice tests, starvation treatment was used as a control towards termite mortality. Fifty termite workers (per cup) were not given any food during test period. The number of replication for starvation treatment is identical to other treatments (seven times for crude extracts and five times for purified extracts) in each feeding test. After the period finished, dead termite were counted to determine the mortality percentage.

#### 2.6.2. Two-Choice Feeding Test Using Extract from *F. radiculosa* Decayed Wood and PDA Media

Two-choice feeding test were performed to assess termite preferences between paper disks treated with decayed wood and PDA media extract. Inside a 200 mL cup (prepared as described in Section 2.6.1), paper disks treated with 100 μL of either decayed wood or PDA extract were placed, 4 cm apart, in a single plastic saucer. One hundred termite workers were introduced into each cup. Test chambers were kept inside an incubator for five days at 25 °C and 70%–80% RH. One *R. speratu*s colony (Kagoshima 2) from Kagoshima and two colonies from Oarai (Oarai 1 and 2) were used in this test. Eight biological replications (eight cups each with 100 *R. speratus*) were performed for each colony. Paper disks were weighed at the end of the test period to assess the mass loss from termite feeding.

### 2.7. Gas Chromatography and Mass Spectrometer Analyses

Chemical analyses of the bioactive fraction in decayed wood and PDA extract was carried out using GC-MS analysis. JEOL MS-600 (JEOL Ltd., Tokyo, Japan) mass spectrometer was coupled with Hewlett-Packard 6890N (Hewlett-Packard, Palo Alto, CA, USA) gas chromatograph was used for GC-MS and helium was used as the carrier gas at 1 mL/min in a constant flow mode. Samples were injected at 200 °C in the split-less mode (sampling time: 0.75 min), separated with a fused silica capillary column (DB-5MS, 25 m × 0.25 mm, 0.25 µm film thickness, Agilent Technology Inc., Santa Clara, CA, USA). Oven temperature was maintained at 50 °C for 3 min, increased to 300 °C at a rate of 5 °C/min, and kept steady for 6 min. The transfer line was kept at 280 °C. Mass spectra were obtained by electron ionization at 70 eV, at a scan speed of 0.29 s with a mass range of 40–600 amu. Major peaks in the chromatogram were compared to those of compounds in the National Institute of Science and Technology (USA) Mass Spectral (NIST MS) library for identification. 

### 2.8. Statisitical Analysis

For feeding tests, treatment and control samples were compared using non-parametric test, Kruskal-Wallis H. The significance level was set at *p* = 0.05. All tests were performed using IBM SPSS Statistics version 22 software (IBM Corp., New York, NY, USA).

## 3. Results

### 3.1. Feeding Deterrent Effect, Toxicity, and Bioactivity of Extracts Obtained from Decayed Wood and PDA Media

**Figure 1:**
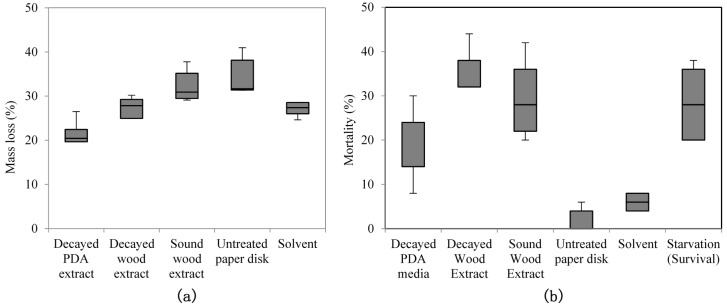
Box plot of (**a**) mass loss and (**b**) mortality in termites caused by feeding on paper disk treated with extracts from decayed wood or potato dextrose agar (PDA) (Kruskal-Wallis H test, *p* < 0.05).

Mass loss differences among the treated paper disks were significantly different (χ^2^(4) = 16.409, *p* = 0.03). The extract obtained from *F. radiculosa*-decayed PDA media was a significantly stronger feeding deterrent than the untreated paper disk (Figure 1). Although the decayed wood extract did not deter termite feeding to the same extent that the PDA media extract did, the mortality caused by it was higher. Based on pairwise comparison, feeding on decayed wood extract led to a similar termite mortality percentage as feeding on sound wood extract. In contrast, feeding on decayed PDA media extract induced a considerably lower mortality that than from feeding on both decayed and sound wood extracts (χ^2^(5) = 21.797, *p* = 0.01). These results suggest that mortality was primarily caused by original or fungus-modified wood components.

The two-choice feeding test was performed to compare the bioactivity of the extracts (Table 1). There were significant differences among the treatments under the Kruskal-Wallis H test (χ^2^(5) = 27.252, *p* < 0.01). Lower mass loss medians were detected for paper disks treated with PDA extract, suggesting that it had a stronger deterrent effect on termite feeding. Mortality was not recorded in this test.

### 3.2. No-Choice Feeding Test Using Purified Extract

**Figure 2:**
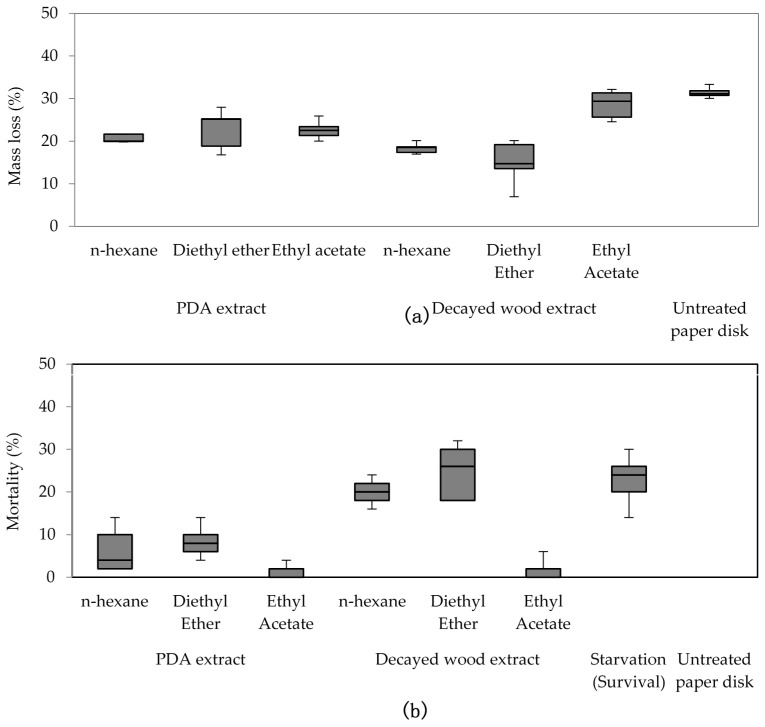
(**a**) Mass loss and (**b**) mortality caused by feeding of purified decayed wood and potato dextrose agar (PDA) extract in a no-choice feeding test (Kruskal-Wallis H test, *p* < 0.05).

To eliminate impurities and determine the bioactive fraction, crude decayed wood and PDA extracts were purified using column chromatography. Differences among treatments were detected by mass loss from termite feeding (χ^2^(6) = 26.575, *p* < 0.01) and termite mortality caused by consumption of purified extract (χ^2^(7) = 31.034, *p* < 0.01). Analysis of mass loss indicated that consumption of decayed wood extract purified with *n-*hexane and diethyl ether was significantly lower than that of extracts purified using other treatments (Figure 2). These extracts also resulted in low survival, indicating that the compound responsible for feeding deterrence or mortality was present in the extracts purified by *n-*hexane and diethyl ether. Although paper disks treated with the diethyl ether fraction from the decayed PDA extract resulted in low termite feeding and slightly higher mortality compared to the other treatments, both the feeding deterrence and the effect on termite survival were inconclusive and not as clear as those of the *n-*hexane or diethyl ether fraction from the decayed wood extract. Thus, the bioactive compound may have been lost from the PDA extract during the purification process.

### 3.3. Analysis of Decayed Wood and PDA Extract with GC-MS

**Figure 3:**
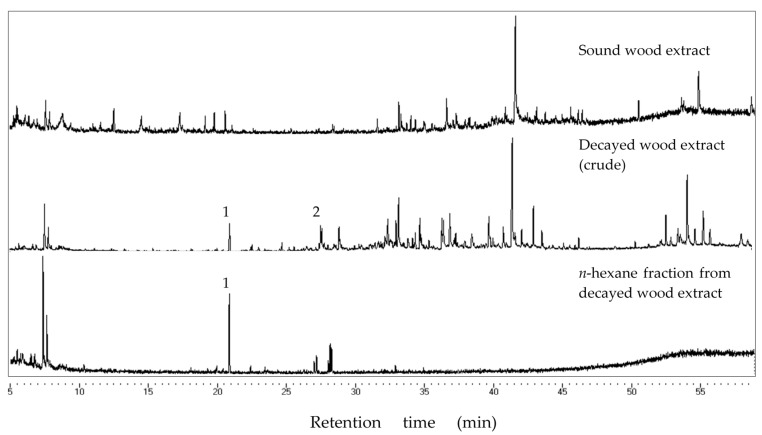
Chromatogram of extracts from sound wood, crude decayed wood, and the *n-*hexane fraction from decayed wood extract. Peak 1 (retention time (RT) 21.11) eluted in the *n-*hexane fraction after chromatography.

**Figure 4:**
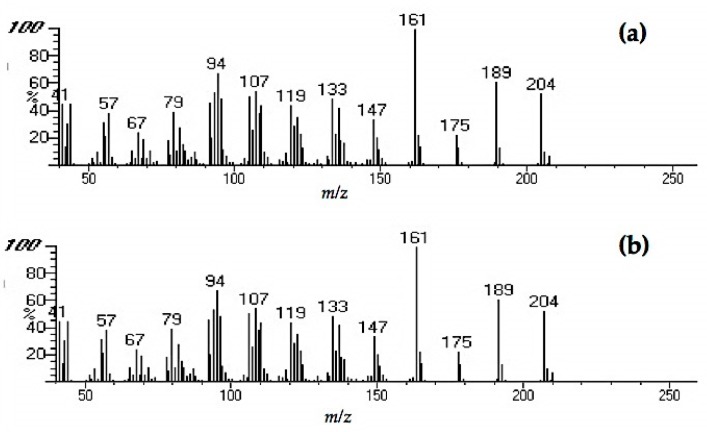
Mass spectra of peak 1 (retention time (RT) 21.11) from (**a**) crude decayed wood extract and (**b**) from *n-*hexane fraction after chromatography.

**Figure 5:**
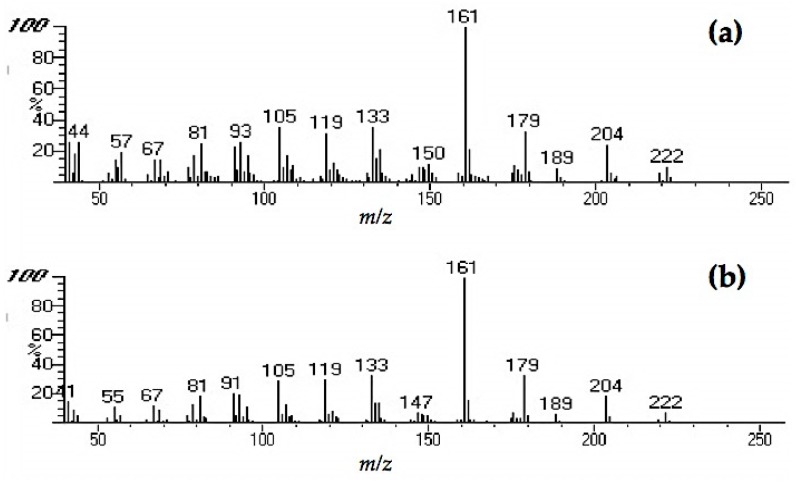
Mass spectra of peak 2 (retention time [RT] 27.72) from (**a**) crude decayed wood extract and (**b**) crude PDA extract.

**Figure 6:**
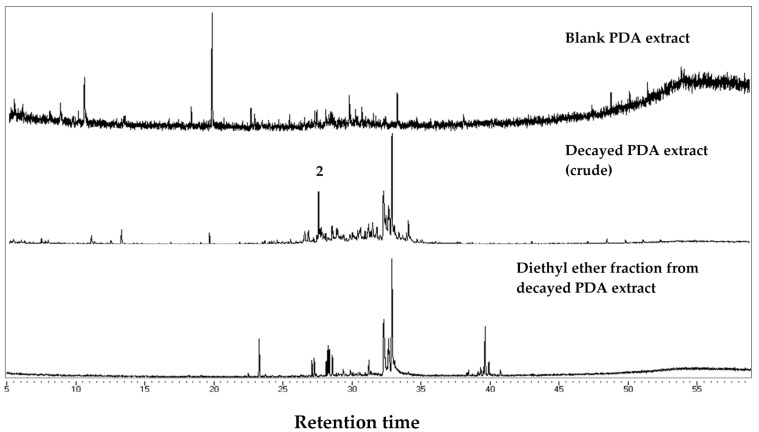
Chromatogram of blank, crude decayed, and diethyl ether fraction from decayed PDA extract. Peak 2 (retention time (RT) 27.72) was also present in the decayed wood extract.

We compared chromatograms from the GC-MS analysis of the sound and decayed wood extracts, as well as the *n*-hexane fraction from the decayed wood extract (Figure 3). Comparisons between the chromatograms of the sound and decayed wood extracts revealed two unique peaks in the decayed wood extract at retention times (RTs) of 21.11 and 27.72 min. After silica gel column chromatography, peak 1 eluted in the *n*-hexane fraction, suggesting that the compound was hydrocarbon. The mass spectrum of peak 1 (Figure 4) showed an ion at *m/z* 204 accompanied by an ion at *m/z* 189 (204-15), which was indicative of a sesquiterpene hydrocarbon (C_15_H_24_). On the other hand, peak 2 (Figure 5) was not detected after the chromatography. Based on the analysis of crude extracts, these compounds share a base ion at *m/z* of 161. By comparing the mass spectra with those in the NIST MS library, peaks 1 and 2 were indicated as longifolene (similarity index 88.9) and hinesol (similarity index 78.2), respectively. Interestingly, peak 2 (RT 27.72) was detected in both decayed wood and PDA media crude extracts, indicating that the origin of peak 2 is from *F. radiculosa* rather than from the wood metabolism, because the compound was also found in non-wood media inoculated with the fungus (Figure 6).

## 4. Discussion

We compared the feeding deterrence and mortality of *R. speratus* fed with crude extracts and fractions from wood and PDA medium decayed by *F. radiculosa* growth, with a view to determine the chemical structure and the origin of the compounds responsible for these effects on the termites.

### 4.1. No- and Two-Choice Feeding of Extract Obtained from Growth Medium Infected with F. radiculosa

Paper disks soaked with both decayed wood and decayed PDA extract had a lower mass loss than did the untreated paper disks, consistent with results from our previous study [9] showing the feeding deterrent in decayed wood was successfully extracted. The PDA extract had a stronger deterrent effect, wherein the feeding is clearly suppressed.

In contrast, the decayed PDA extract resulted in a lower mortality compared with that of the decayed wood extract in a no-choice feeding test (Figure 1); this outcome might be due to the absence of wood extractives. The fact that the mortality did not significantly differ between extracts from sound wood and decayed wood implies the role of the wood material in lowering the termite survival. Although a mortality effect was not apparent for the PDA extract, the deterrent effect of this extract may act as a mortality cofactor. Reduced consumption caused by a feeding deterrent effect can reduce fitness, thereby exposing termites to a variety of factors that affect mortality [10].

Fungal secondary metabolites, such as volatile organic compounds (VOCs), can modulate insect feeding behavior. In certain conditions, fungal secondary metabolites can adversely affect termite feeding on rotting wood. Similar to allelopathy mechanisms in plants, the export of toxic secondary metabolites might drive competition between insects and fungi [11] that feed within the same niche. Deterrents released by *F. radiculosa* initially affected the pre-ingestion stage in termite feeding, specifically during orientation or host selection. In the pre-ingestion stage (food recognition followed by acceptance or rejection), feeding deterrents interfere with sensors located in the mouthparts, food canal, and antennae of the insects [12]. The deterrent interferes with the termite’s gustatory and olfactory sensation via blocking impulse transmission towards neural control circuits. In the absence of a feeding stimulant or the presence of a deterrent, termites will perceive the interference as a sign that the compound is inedible or nutritionally inadequate, leading to the rejection of decayed wood as a food source.

Interestingly, in the two-choice feeding test (Figure 2), two colonies displayed distinctive preferences towards decayed wood extract over decayed PDA. The data suggest that there may be a stronger feeding stimulant, masking the detection of a deterrent and/or toxic compound. This possibility is not unexpected; termites were found to prefer a cellulosic disk treated with *Pinus densiflora* extract and exhibited continuous feeding stimulation in the presence of this extract [13].

### 4.2. Mass Loss and Mortality Caused by the Feeding of Extract Obtained from Chromatographed Decayed wood and PDA and Analysis by GC-MS

The *n*-hexane and diethyl ether fractions deterred termite feeding and induced termite mortality. The fact that bioactivity of the extract was retained after chromatography indicates the non-polar nature of the feeding deterrent. Purified extract with the more polar ethyl acetate did not show any notable feeding deterrence or mortality compared with the controls.

Multiple components were involved in the deterrence of termite feeding from the decayed wood extract. Peak 1 (RT 21.11) remained in the *n*-hexane fraction of decayed wood and was undetected in decayed PDA media (Figure 3); thus, this compound is suspected to be a wood modification product synthesized during fungal decay. Meanwhile, peak 2 (RT 27.72) was detected in the chromatogram from crude decayed samples (Figure 6), indicating that peak 2 is a compound released by *F. radiculosa* rather than a metabolized wood product.

The mass spectra of peaks 1 and 2 indicated that these compounds are sesquiterpenes. Peak 1 (Figure 4), which the NIST MS library indicated as longifolene, plays an important role in wood resistance to termite attacks. Xu et al. [14] suggested that this is one of the key components of *Pinus massoniana* resistance to *C. formosanus* attacks. Studies examining the function of hinesol (match of peak 2, RT 27.72) shown in Figure 5, as a termite feeding deterrent have not been conducted to date to the best of our knowledge, although several studies have already documented the association of other sesquiterpenes with insect behavior.

The differences in the amount and type of feeding deterrents produced by decayed wood and PDA media may be strongly linked with carbon and nitrogen as nutrient sources for *F. radiculosa*. Unlike fungi growing on soft wood, fungi grown in PDA medium are nourished with only a single sugar source. Secondary metabolite production depends on the composition of the culture medium and culture conditions. For example, sesquiterpene production by the cultured basidiomycete *Lentinus lepideus* varied depending on the type of sugar-enriched standard culture media. The sugar type influenced both the sesquiterpene increase and the decrease of volatile cinnamic acid derivatives; thus, the production of secondary metabolites in the form of VOCs is dependent on the fungal nutrient source [15].

## 5. Conclusions

Extracts from fungi-decayed PDA media inhibited *R. speratus* feeding, indicating that the deterrent substance originated from *F. radiculosa* rather than the wood decay process. Despite the stronger feeding deterrent effect of the crude PDA extract, its effects differed from those of decayed wood extract. Conceivably, decayed wood extract may contain two bioactive compounds that deter feeding and are toxic to termites. Chromatography easily removed the decayed PDA media–derived bioactive compound, causing the purified extract to lose its deterrent characteristics and weakly suppress termite feeding. According to the NIST MS match, the bioactive compounds were indicated to be sesquiterpenes—the decayed wood extract contained two bioactive suspect compounds, longifolene (similarity index 88.9) and hinesol (similarity index 78.2), while the PDA extract contained only hinesol. The *F. radiculosa* growth medium appears to be primarily responsible for the differences in the bioactive compound produced. Future studies on termite-fungi interactions should aim to further investigate the effects of *F. radiculosa* growth media and variations in feeding deterrents produced.

## Figures and Tables

**Table 1:** Mass loss medians of two-choice feeding test between crude decayed wood and PDA media extract in test colonies.

Colony	Decayed Wood Extract (%)	PDA Extract (%)
Colony 1 (Kagoshima 2)	21.19	0
Colony 2 (Oarai 1)	34.60	19.02
Colony 3 (Oarai 2)	59.60	3.42

Kruskal-Wallis H test, *p* < 0.05.
